# Chronic Polystyrene Microplastic Exposure Reduces Testosterone Levels in Mice through Mitochondrial Oxidative Stress and BAX/BCL2-Mediated Apoptosis

**DOI:** 10.3390/toxics12080561

**Published:** 2024-08-01

**Authors:** Yi Liu, Xiaomin Li, Ying Xiong

**Affiliations:** 1 Department of Anesthesiology, Zhongnan Hospital of Wuhan University, Wuhan 430071, China; 2 Reproductive Medicine Center, Tongji Hospital, Tongji Medicine College, Huazhong University of Science and Technology, Wuhan 430030, China; 3 Department of Pharmacology, Taikang Medical School (School of Basic Medical Sciences), Wuhan University, Wuhan 430071, China

**Keywords:** microplastics, oxidative stress, apoptosis, Leydig cell, testosterone

## Abstract

Microplastics (MPs) have emerged as a major environmental issue. They have been found to cause significant reproductive toxicity and lower testosterone levels in adult males, though the exact mechanisms remain unclear. In this study, C57bl/6 mice were orally exposed to saline or varying doses (0.25, 0.5, and 1 mg/day) of 5 μm polystyrene MPs (PS-MPs) for 4 weeks, and TM3 mouse Leydig cells were treated with different concentrations of PS-MPs. Our results found that exposure to PS-MPs significantly reduced testosterone levels and impaired the synthesis function of testicular steroids. In vitro, PS-MPs reduced steroid synthesis in Leydig cells. Treatment with PS-MPs significantly increased the apoptosis rate and BAX/BCL2 ratio in Leydig cells. Additionally, GSH-px and SOD activities decreased, while MDA levels increased, along with a rise in mitochondrial ROS. In conclusion, chronic PS-MP exposure reduced testosterone levels in mice through mitochondrial oxidative stress and BAX/BCL2-mediated apoptosis. This study offers new insights into the health risks posed by MPs.

## 1. Introduction

Due to the significant growth of the plastic manufacturing industry, plastic waste has surged. In 2020, the world produced nearly 370 million tons of plastic [1]. Consequently, a new type of environmental pollutant called microplastics (MPs) has emerged. These particles are primarily created from the degradation of larger plastic fragments. In recent years, pollution problems associated with MPs have gradually emerged as a pervasive global environmental threat. It is known that MPs can be found in various places such as water, sea salt, aquatic foods, and industrial abrasives [2,3,4]. In addition, multiple fragments of MPs have been discovered in human placental samples through the use of Raman Microspectroscopy by researchers [5]. And inadvertently consuming aquatic organisms that have accumulated microplastics poses a potential threat to human health [6]. People are exposed to various types of MPs; however, the impact of these particles on our health remains unclear, and consideration should be given to the prospective health effects of MPs.

MPs are easily absorbed by animals and persistently accumulate due to their small size and durability. Previous research has shown that MPs accumulate in organisms, resulting in various harmful effects. Wei et al. demonstrated that MPs interfere with lipid metabolism in human stem cell-derived liver organoids, leading to hepatotoxicity [7]. Additionally, chronic exposure to MPs has been linked to hepatotoxicity and gut microbiota dysbiosis in mice, as reported by Chen et al. [8]. Furthermore, studies have observed intestinal barrier dysfunction, inflammation, hepatic lipid disorders, and liver fibrosis in mice treated with MPs [9,10,11]. Moreover, MPs can penetrate the blood–brain barrier, triggering microglial activation and neuroinflammation, crucial for synaptic plasticity and memory [12]. These findings underscore the potential threat of MPs to organism health.

Testosterone, a steroid hormone originating from the Leydig cells of the testes, is crucial for maintaining male sexual function, bone health, and overall quality of life. Recent studies have shown that exposure to PS-MPs induces decreased testosterone levels, leading to male reproductive dysfunctions in mice [13,14,15]. However, prior studies have mainly focused on observations, leaving the precise mechanism by which MPs disrupt testosterone synthesis unclear. This study aimed to utilize mice as an animal model to validate the impact of MPs on testosterone synthesis function. The levels of BAX/BCL2 apoptosis and oxidative stress in the testes were further observed. Additionally, TM3 mouse Leydig cells were employed in vitro to delve into the underlying mechanisms. These results could provide a basis for studies on the effect of MPs on male reproductive health and offer novel perspectives on the potential health hazards associated with MPs.

## 2. Materials and Methods

### 2.1. Chemicals and Reagents 

The suspensions of polystyrene microplastics (PS-MPs) with a particle size of 5 μm (1.0% *w*/*v*, 10 mL) were sourced from Tianjin Baseline ChromTech Research Centre in Tianjin, China. Serum CORT levels were measured using an ELISA kit (cat. NO. KGE009, R&D Systems Inc., Minneapolis, MN, USA). Gibco (Thermo Fisher Scientific, Scoresby, Australia) provided DMEM/F12 and fetal bovine serum (FBS), while Invitrogen (Carlsbad, CA, USA) supplied penicillin and streptomycin. Antibodies for β-Actin (AC006), BAX (A19684), BCL2 (A0208), COX2 (A1253), iNOS (A14031), and steroidogenic acute regulatory protein (StAR) (A16432), as well as conjugated goat anti-rabbit IgG (AS007), were obtained from ABclonal Technology Co., Ltd. (Wuhan, China). The FITC and Cy3 conjugated goat anti-mouse IgG antibodies (GB22301 and GB21301) were acquired from Servicebio Inc. (Wuhan, China). The protease and phosphatase inhibitors, namely Complete Mini and PhosSTOP, were acquired from Roche. The TRIzol reagent was obtained from Thermo Fisher Scientific Co., Ltd. (Waltham, MA, USA). The BCA Protein Assay Kit (P0011) was obtained from Beyotime Biotechnology, a company based in Shanghai, China. The MTS [3-(4,5-dimethylthiazol-2-yl)-5-(3-carboxymethoxyphenyl)-2-(4-sulfophenyl)-2H-tetrazolium] assay kit was purchased from Cayman Chemical Co. (Ann Arbor, MI, USA). The reverse transcription and real-time quantitative polymerase chain reaction (RT-qPCR) kits were acquired from Takara Biotechnology Co., Ltd. (Dalian, China). All other chemicals and reagents used were of analytical grade.

### 2.2. Animals and Treatment

Male C57BL/6 mice aged six weeks were obtained from the Wuhan University Animal Centre in Wuhan, China. Mice were raised in a constant temperature environment and acclimatized for one week to 12-h light–dark cycles. The animals had unrestricted access to food and water. The Animal Experiment Committee at Wuhan University granted approval for all animal procedures (approval NO. WP20230135). Before being utilized, the 5 µm microplastic particle was evenly spread out in deionized water and subjected to supersonic wave vibration for a duration of 20 min. Various concentrations of PS-MPs or sterile water were orally gavaged once daily to animals. The animals were randomly divided into four groups, and each group contained eight mice: the control group, the MP(L) group (0.25 mg/d PS-MPs), the MP(M) group (0.5 mg/d PS-MPs s), and the MP(H) group (1 mg/d PS-MPs). Treatment lasted for 4 weeks. The MP groups ingested deionized water containing microplastics, whereas the control group ingested deionized water without any additional substances. The doses of PS-MPs and the frequency of oral gavage were determined based on a previous animal model study [13,16,17]. The body weight was determined three times per week. The mice were then humanely euthanized via CO_2_ exposure at the end of the experiments, and the serum was obtained by subjecting the sample to centrifugation with a force of 1500× *g* for a duration of 10 min. Subsequently, it was preserved at a temperature of −80 °C until examination. In addition, testes were fixed for 48 h with 4% paraformaldehyde for histological sectioning, and others were frozen at −80 °C for further analysis.

### 2.3. Characterization of PS-MPs

The morphology of PS-MPs was examined using a field emission scanning electron microscope (SEM) (Hitachi, Japan). Surface functional groups of the polystyrene microspheres were analyzed with Raman microscopy and ATR-FTIR spectroscopy (InVia; Renishaw, Inc., West Dundee, IL, USA). SEM analysis (Figure S1A) revealed that the PS-MPs had a uniform spherical shape. The Raman spectra (Figure S1B) and ATR-FTIR spectrogram (Figure S1C) both verified that the microspheres were composed of polystyrene.

### 2.4. Immunohistochemistry (IHC) and Immunofluorescence (IF) Analysis

IHC and IF experiments were performed by slightly modifying the previously described method [18]. In brief, the right testicles of three animals from each group were selected, fixed in 4% paraformaldehyde, embedded in paraffin, and cut into 4 μm sections along their longitudinal axis. The final results were obtained after a series of steps such as dewaxing, antigen repair, blocking, incubation with primary antibody, incubation with secondary antibody, and color rendering. Images were obtained using a Leica DMRA microscope (Leica, Heidelberg, Germany). And image-Pro Plus software (version 6.1, Media Cybernetics, Silver Spring, MD, USA) was used to analyze the total area and number of positive cells in each field, and the average values of the five fields were used to compute the data for each sample. Quantification was concealed from researchers during the experiment and analysis of data. 

### 2.5. Cell Culture and Treatment

TM3 mouse Leydig cells were sourced from Procell Life Science & Technology Co. in Wuhan, China. The cells were cultured according to the previous method [18]. In this study, cells were exposed to varying concentrations of PS-MPs (0, 0.05, 0.1, 0.2, 0.4, and 0.8 mg/mL) for 24 h and then collected for further analysis. All experiments were independently repeated twice, with the number of wells within each independent experiment ranging from 3 to 6. 

Firstly, the cytotoxic effects of PS-MPs were assessed using the MTS assay. TM3 cells were seeded into a 96-well tissue culture plate at a concentration of 2 × 10^5^ cells/mL, 150 μL/well. After a certain period of cell culture with TM3, 20 μL of MTS reagent was added to each well, and the absorbance was measured at 490 nm using a microplate reader after 1 h of MTS incubation. This study used PS-MP concentrations of 0, 0.05, 0.1, 0.2, 0.4, and 0.8 mg/mL to treat TM3 cells for at least 24 h. 

### 2.6. Transmission Electron Microscopy (TEM)

Following the procedure described by Wang et al. [17], testis tissues were embedded in EPON resin, sliced to a thickness of 60 nm, and analyzed using transmission electron microscopy (TEM). The samples underwent a series of steps: they were washed three times with 0.1M cacodylate for 15 min each, then treated with 1% osmium tetroxide for 1–2 h, followed by another three washes with 0.1M cacodylate for 15 min each. Subsequently, they were sequentially washed with 70%, 80%, 90%, 95%, and 100% ethanol for 15 min each. Finally, they were incubated in propylene oxide for 10 min.

### 2.7. Annexin V/PI Staining and Flow Cytometry 

We evaluated the proportion of apoptotic cells that were exposed to different concentrations of PS-MPs by utilizing Annexin V/PI labelling and flow cytometry. The detection kit was used following the manufacturer’s instructions. Briefly, cells were trypsinized with EDTA-free trypsin, washed twice with cold PBS, and resuspended in 400 μL binding buffer. Annexin V and PI were added, and the mixture was incubated on ice for 15 min in the dark. The apoptosis rate of TM3 cells was then analyzed using flow cytometry.

### 2.8. Evaluation of Mitochondrial Membrane Potential (MMP)

MMP was assessed using the JC-1 fluorescent probe following a previously described method [19]. The detection kit was utilized according to the manufacturer’s instructions. Excitation and emission wavelengths of 514 nm and 529 nm were used to detect the monomeric form of JC-1, while 585 nm and 590 nm were used for detecting JC-1 aggregates. Red fluorescence emission indicates the presence of JC-1 polymer in the cell, signifying a higher mitochondrial membrane potential. Conversely, green fluorescence emission indicates the presence of JC-1 monomers in the cell, indicating a decrease in mitochondrial membrane potential.

### 2.9. TUNEL Assay

The TUNEL detection kit, containing terminal deoxynucleotidyl transferase (TdT), was rapidly prepared according to the manufacturer’s instructions before being used. After rinsing with PBS, the sections were stained with DAPI as a counterstain. The apoptotic cells in the sections were observed using a microscope.

### 2.10. Total RNA Extraction and Quantitative Real-Time PCR (RT-qPCR)

Testicular tissue or cells were extracted using TRIzol Reagent according to the manufacturer’s guidelines. The process of reverse transcription was performed on the total RNA using a cDNA synthesis kit to generate the first strand of cDNA. Subsequently, RT-qPCR was performed using the SYBR Green qPCR Master Mix Kit and an ABI StepOnePlus cycler (Applied Biosystems, Foster City, CA, USA). Primer sequences for the genes investigated in rats and mice are provided in Table S1. The cycle threshold (Ct) was measured, and the relative gene expression was computed using the 2^(ΔΔCt)^ method. The expression was normalized to GAPDH expression and used as a quantitative control.

### 2.11. Measurement of SOD, MDA, and GSH-px

The xanthine oxidase method from the standard test kit (Nanjing Jiancheng Bioengineering Institute, China) was used to measure the activity of superoxide dismutase (SOD) and glutathione peroxidase (GSH-px) in both sera and culture media. The assessment of lipid peroxidation was conducted by quantifying the levels of malondialdehyde (MDA) through the utilization of the commercially endorsed thiobarbituric acid (TBA) technique. The absorbance was quantified using a microplate reader (Benchmark; Bio-Rad Laboratories, Inc., Hercules, CA, USA) in accordance with the manufacturer’s guidelines.

### 2.12. Analysis of Testosterone

Testosterone levels were measured using I125-testosterone Coat-A-Count RIA kits through a radioimmunoassay (RIA) following the manufacturer’s protocol.

### 2.13. Statistical Analysis

Data analysis was conducted using SPSS 19 (SPSS Science Inc., Chicago, IL, USA) and Prism 8.0 (Graph Pad Software, La Jolla, CA, USA). Quantitative data are presented as mean ± S.E.M. A two-tailed Student’s t-test was used to compare the control and treatment groups. For experiments involving more than two groups, data were analyzed using ANOVA followed by Tukey’s post hoc test. The threshold for statistical significance was established at a *p*-value of less than 0.05.

## 3. Results

### 3.1. Testicular Morphology and Steroid Synthesis Function

**Figure 1:**
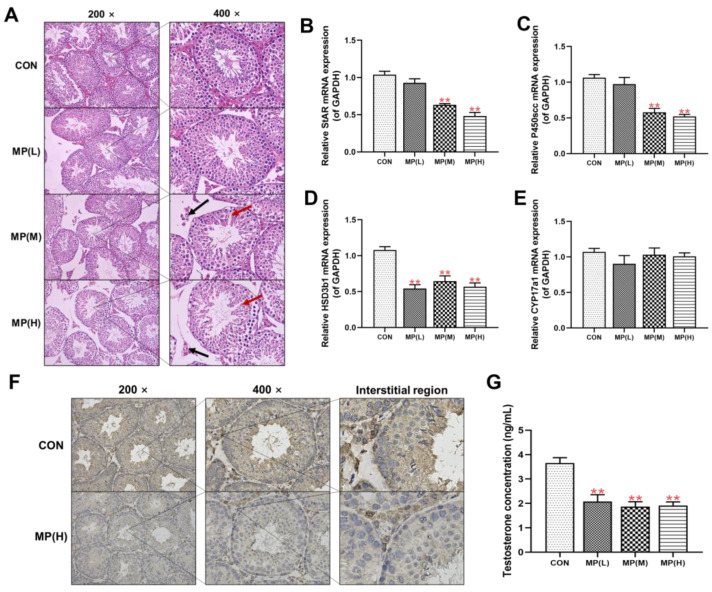
The impact of PS-MPs on testicular morphology and steroid synthesis function. (**A**) Testicular morphology was assessed using HE staining (200×, 400×, n = 3). (**B**–**E**) Expression levels of StAR, P450scc, HSD3b1, and CYP17a1 genes were measured by RT-qPCR (n = 10). (**F**) StAR protein expression was examined through IHC staining (200×, 400×, n = 3). (**G**) Serum testosterone concentration was quantified (n = 10). Values are presented as means ± S.E.M., ** *p* < 0.01 vs. control. PS-MPs refers to polystyrene microplastics; HE to hematoxylin and eosin; WB to Western blot; IOD to integral optical density; RT-qPCR to real-time quantitative polymerase chain reaction; IHC to immunohistochemistry; StAR to steroidogenic acute regulatory protein; P450scc to cytochrome P450 cholesterol side chain cleavage; HSD3b1 to hydroxysteroid 3-beta dehydrogenase 1; and CYP17a1 to cytochrome P450 family 17 subfamily A member 1.

Initially, the effects of various doses of PS-MPs on testicular morphology and testosterone synthesis function in male mice were assessed. Compared to the control group, the testes in the MP(M) and MP(H) groups exhibited pathological changes, such as narrowing of the interstitial area and abnormal structure of seminiferous tubules (Figure 1A). Pathological changes in the interstitial and seminiferous tubules are indicated by black and red arrows, respectively. The mRNA expression levels of key enzymes involved in testosterone synthesis (including StAR, P450scc, and HSD3b1) in the testes decreased in a dose-dependent manner (Figure 1B–E). Immunohistochemistry (IHC) results showed significantly lower distribution and expression of StAR, a crucial enzyme for testicular testosterone synthesis, in testicular interstitial cells in the MP(H) group (Figure 1F). Additionally, serum testosterone levels significantly decreased in the PS-MP treatment group (Figure 1G). After chronic exposure to PS-MPs, testicular morphological development and steroid synthesis function were impaired.

### 3.2. Testosterone Synthesis Function in Testicular Leydig Cells 

**Figure 2:**
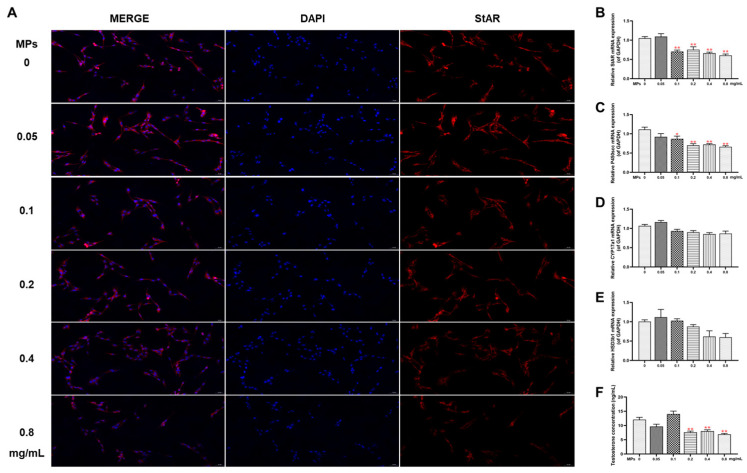
The impact of PS-MPs on steroid synthesis function in testicular Leydig cells. (**A**) StAR protein expression detected via IF staining (StAR stained in red, 200×, n = 3). (**B**–**E**) Expression of StAR, P450scc, HSD3b1, and CYP17a1 genes assessed by RT-qPCR (n = 6). (**F**) Measurement of testosterone concentration (n = 6). Means ± S.E.M., * *p* < 0.05, ** *p* < 0.01 vs. control. PS-MPs refers to polystyrene microplastics; RT-qPCR to real-time quantitative polymerase chain reaction; IF to immunofluorescence; StAR to steroidogenic acute regulatory protein; P450scc to cytochrome P450 cholesterol side chain cleavage; HSD3b1 to hydroxysteroid 3-beta dehydrogenase 1; and CYP17a1 to cytochrome P450 family 17 subfamily A member 1.

TM3 mouse Leydig cells were treated with PS-MPs at concentrations of 0, 0.05, 0.1, 0.2, 0.4, or 0.8 mg/mL for 24 h to further assess the damage caused by PS-MPs to the testes. The MTS assay was employed to assess cell viability, revealing no notable differences among the treatment groups except following 72 h of PS-MP treatment (Figure S2A–C). IF analysis revealed a gradual decrease in the fluorescence intensity of StAR with increasing concentrations of PS-MPs (Figure 2A). RT-qPCR results demonstrated that mRNA levels of StAR and P450scc in the testes significantly decreased in a concentration-dependent manner (Figure 2B–E). Furthermore, testosterone levels gradually decreased with increasing PS-MP concentration (Figure 2F).

### 3.3. Cell Apoptosis in Testicular Leydig Cells 

**Figure 3:**
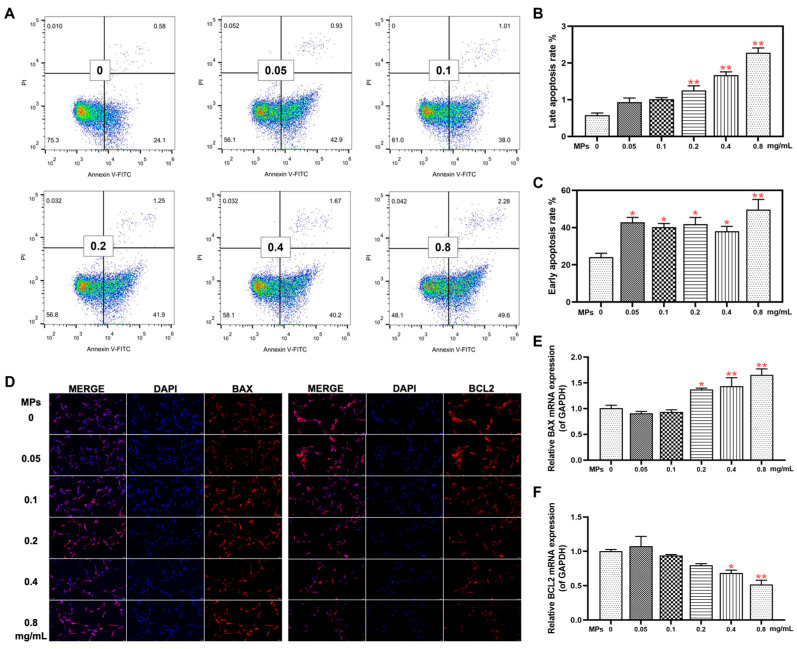
The effect of PS-MPs on cell apoptosis in Leydig cells of the testes. (**A**–**C**) Cell apoptosis was detected using flow cytometry, with analysis of early and late apoptosis rates (n = 3). (**D**) IF staining was conducted to assess BAX and BCL2 protein expression (BAX and BCL2 stained in red, 200×, n = 3). (**E**,**F**) RT-qPCR was employed to evaluate the expression of BAX and BCL2 genes (n = 6). Means ± S.E.M., * *p* < 0.05, ** *p* < 0.01 vs. control. PS-MPs refers to polystyrene microplastics; WB to Western blot; IOD to integral optical density; RT-qPCR to real-time quantitative polymerase chain reaction; IF to immunofluorescence; BAX to BCL2-associated X protein; and BCL2 to BCL2 apoptosis regulator.

Our previous research has shown that PS-MPs induce a decrease in sperm quality by activating spermatogonium mitochondrial oxidative stress and apoptosis [20]. The mechanism of testicular Leydig cell injury was further investigated in this study. Cell apoptosis was assessed using Annexin V/PI staining, revealing a significant concentration-dependent increase in both early and late apoptosis percentages (Figure 3A–C). Meanwhile, IF intensity and mRNA expression levels of BAX increased in a concentration-dependent manner, whereas a concentration-dependent reduction in BCL2 was observed (Figure 3D–F). 

### 3.4. Mitochondrial Function in Testicular Leydig Cells

**Figure 4:**
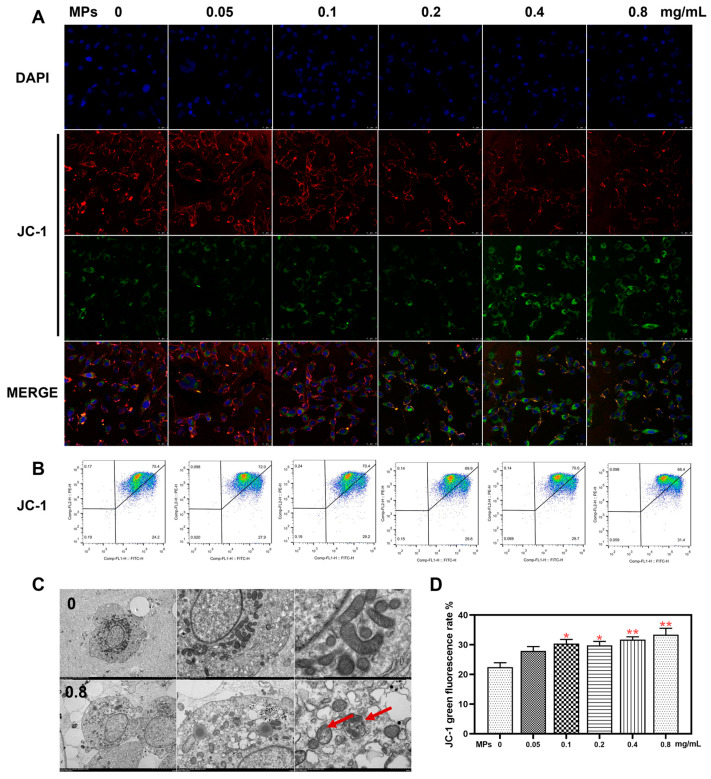
The effect of PS-MPs on mitochondrial function in Leydig cells of the testes. (**A**) Detection of mitochondrial membrane potential using the JC-1 fluorescent probe (400×, n = 3). (**B**) Assessment of mitochondrial membrane potential via flow cytometry. (**C**) Evaluation of mitochondrial morphology using TEM (n = 3). (**D**) Analysis of JC-1 fluorescence by flow cytometry (n = 3). Means ± S.E.M., * *p* < 0.05, ** *p* < 0.01 vs. control. PS-MPs refers to polystyrene microplastics; TEM to transmission electron microscope.

Following treatment with PS-MPs, there was a gradual decrease in red fluorescence intensity and a corresponding increase in green fluorescence intensity with increasing concentration (Figure 4A). Flow cytometry analysis revealed a concentration-dependent increase in the positive rate of JC-1 green fluorescence (Figure 4B,D). The TEM investigation revealed that the mitochondrial cristae in the experimental group appeared to be fuzzy or disorganized in comparison to the normal control group, and the number of autophagosomes increased in the group that received a dosage of 0.8 mg/mL of PS-MPs (Figure 4C). The red arrow indicates damaged mitochondria. 

### 3.5. Oxidative Stress in Testicular Leydig Cells

**Figure 5:**
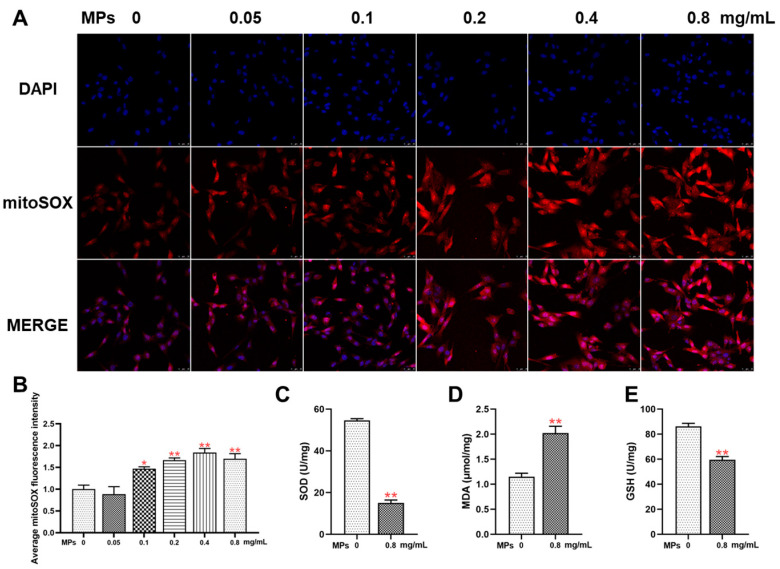
The impact of PS-MPs on oxidative stress in testicular Leydig cells. (**A**) Quantification of mitochondrial reactive oxygen species (ROS) levels with the application of Mito SOX labeling (ROS stained in red, 400×, n = 3). (**B**) Quantitative evaluation of fluorescence intensity data obtained from MitoSOX staining (n = 3). (**C**–**E**) Evaluation of antioxidant activity of SOD and GSH-px, and measurement of MDA concentration (n = 6). Means ± S.E.M., * *p* < 0.05, ** *p* < 0.01 vs. control. PS-MPs refers to polystyrene microplastics; ROS to reactive oxygen species; SOD to superoxide dismutase; GSH-px or GSH to glutathione peroxidase; and MDA to malonaldehyde.

Cells treated with different concentrations of PS-MPs exhibited higher levels of mitochondrial ROS compared to the control group (Figure 5A,B). Additionally, exposure to 0.8 mg/mL PS-MPs resulted in increased lipid peroxidation product MDA levels, accompanied by a decrease in the activity of the antioxidant enzymes GSH-px and SOD (Figure 5C–E). 

## 4. Discussion

Many studies have documented the negative impacts of MPs on marine life, including the induction of gastrointestinal and liver damage in aquatic species [21]. However, due to human activities, land-based MP pollution is escalating much faster than marine pollution [22]. Mammals unavoidably encounter MPs through the process of consuming them or breathing them in. Assessing toxicity requires careful consideration of the dose and particle size in MP exposure [13]. A study by Nor et al. [23] suggested that the intake of microplastics can cumulatively reach up to 40.7 (90% CI, 0.8–9.85 × 10^3^) ng/person in an irreversible manner. Zhang et al. [24] measured and identified microplastics in the residual fecal matter of 26 male college students using Fourier Transform Infrared Microspectroscopy and reported that the summed mass of all microplastic particles per participant ranged from 0.01 to 14.6 mg. Additionally, a recent meta-analysis from fifty-nine publications indicates that humans could ingest 0.1–5 g of microplastics weekly through various exposure routes [25]. There are significant variances in the reported concentrations of microplastics in the human body due to differences in research methods and measurement techniques. These methodological challenges underscore the need for standardizing methods for determining microplastics [26]. In our study, C57BL/6 mice were exposed to ultrapure water or different doses (0.25, 0.5, and 1 mg/d) of PS-MPs (a standard microplastic product) for 4 weeks to assess their biological toxicity. This exposure level corresponded to mice weighing 25 mg being subjected to daily doses of 10, 20, and 40 mg/kg of PS-MPs. The exposure concentrations chosen for this study were primarily based on the levels to which humans are exposed via consumer products [27], and integrated the most current toxicological research [28]. Following the guidelines for converting animal doses to human equivalents [29], the maximum dose of PS-MPs (40 mg/kg) utilized in the mice in this study corresponds to 3.244 mg/kg in humans. Therefore, for an adult weighing 60 kg, this dosage translates to an intake of 194.64 mg/person/day. This value falls within the estimated mass range for microplastic exposure [25]. Given these findings, we acknowledge the challenges in accurately quantifying the concentration of microplastics in humans. We anticipate that as research in this field advances and measurement techniques improve, our understanding of this issue will become clearer. The distribution of MPs in various organs is also contingent upon the size of the particles [30]. Research has shown that 5 μm diameter PS-MPs have a higher tendency to collect in different organs of mice, such as the liver and gut, compared to PS-MPs of other diameters [31]. The bioaccumulation of 5 µm PS-MPs in tissue was twice as high as that of 20 µm PS-MPs [32]. During this experiment, we selected a moderate particle size of 5 μm for PS-MPs and observed the effects of chronic exposure over 4 weeks. Thus, the animal model used in this study is suitable for investigating the impact of PS-MPs on organism function.

We selected concentrations of 0, 0.05, 0.1, 0.2, 0.4, and 0.8 mg/mL for our in vitro studies based on previous research on the toxicity of microplastics at the cellular level [17]. These studies have demonstrated that these concentrations can be relevant for observing potential biological effects without causing significant toxicity, as confirmed by our MTS assay results which showed no significant cytotoxicity at these levels. While direct evidence from in vivo experiments or epidemiological studies on the concentrations of microplastics circulating in human blood is still limited, Leslie et al. [33] have detected microplastics in human blood at concentrations ranging from 1.6 to 4.8 µg/mL. Our chosen concentrations aim to cover a range of plausible exposure scenarios, providing a comprehensive understanding of potential cellular effects. We acknowledge that the concentrations used in our cell experiments are relatively high. Meanwhile, real-world exposures to microplastics encompass a variety of plastic sizes, shapes, and types. Consequently, exposure to a single plastic size and type (e.g., 5 µm polystyrene beads) oversimplifies the complexity of real-world exposures. However, they are still valuable for toxicological research, as they help us understand the potential upper limits of microplastic exposure and its effects on cellular functions.

The testis, the principal reproductive organ in males, plays a crucial role in ensuring the quality of sperm and the proper functioning of the reproductive system. Current studies indicate that PS-MPs can impair sperm quality [13,30,34,35,36]. Findings show that exposure to PS-MPs reduces sperm count and increases the rate of sperm deformity. Meanwhile, testosterone synthesis and secretion are primarily carried out by Leydig cells in the testis, which are crucial for spermatogenesis, including maintaining the blood–testis barrier, meiosis, and sperm release [37]. Testosterone production in Leydig cells involves the conversion of cholesterol into intermediate steroids, regulated mainly by StAR and P450scc enzymes. A decrease in testosterone levels can directly affect male reproductive function. Although numerous studies have highlighted the effects of PS-MPs on testosterone levels and testicular steroid synthesis, previous research on the reproductive toxicity of PS-MPs has often lacked comprehensive in vitro experiments. One study has shown that PS-MPs reduce testosterone levels by down-regulating the LHR/cAMP/PKA/StAR pathway in primary Leydig cells [30]. In our study, we treated TM3 mouse Leydig cells with varying concentrations (0, 0.05, 0.1, 0.2, 0.4, and 0.8 mg/mL) of PS-MPs to explore their mechanisms of damage. Our findings revealed that PS-MPs entered Leydig cells in vivo, resulting in a significant decrease in testosterone levels and steroid synthesis function. Similarly, in vitro experiments exhibited a dose-dependent reduction in steroid synthesis in Leydig cells exposed to PS-MPs. Therefore, PS-MPs directly damage the testosterone synthesis function of Leydig cells.

Oxidative stress occurs when the balance between prooxidants and antioxidants in cells is disrupted [38]. This leads to severe oxidative damage and ultimately impairs cell function. To counter oxidative stress, cells utilize their intrinsic antioxidant mechanisms. SOD and GSH-px work together to convert peroxides into less harmful hydroxyl compounds, thereby preventing the accumulation of ROS. MDA, a byproduct of lipid peroxidation, functions as an indirect marker of ROS levels and lipid peroxidation damage [39]. Mitochondria play a crucial role in the formation of oxygen free radicals due to electron transfer during mitochondrial respiration [40]. As the primary producers of ROS, mitochondria are also the main targets of increased ROS exposure, which can lead to fatal consequences such as apoptosis [41,42]. Apoptosis, the process of cell death, is controlled by multiple variables. Among these factors, mitochondria have a crucial function in receiving and delivering signals that induce cell death, such as oxidative stress and DNA damage. This enables mitochondria to regulate the pathways leading to cell death [43,44]. The apoptotic process is regulated by BCL2 family proteins and is associated with the structure of mitochondria, membrane potential, and other functions [45]. In summary, oxidative stress contributes to organ damage induced by various environmental toxins and drugs, and induces apoptosis through mitochondria-dependent pathways.

Our previous research found that sperm quality declined after exposure to PS-MPs, with testes showing pathological changes such as abnormal spermatogenic tubule development and inhibited spermatogonium function. TUNEL staining fluorescence intensity and the BAX/BCL2 ratio increased. PS-MP exposure impaired mitochondrial morphology, decreased GSH-px and SOD activity, and raised MDA levels. In vitro, PS-MPs increased spermatogonium apoptosis, decreased mitochondrial membrane potential, damaged mitochondrial morphology, and elevated mitochondrial reactive oxygen species, leading to oxidative stress [20]. However, the effect of oxidative stress on steroid synthesis dysfunction in male mice caused by PS-MPs requires further exploration. This study observed that, in vitro, although PS-MPs did not directly affect cell viability, both early and late apoptosis rates of Leydig cells and the BAX/BCL2 ratio significantly increased. Following PS-MP treatment, mitochondrial membrane potential decreased, mitochondrial morphology was damaged, and mitochondrial ROS levels increased. Furthermore, the activities of GSH-px and SOD decreased, while MDA levels rose. These findings suggest that PS-MPs increased mitochondrial ROS levels and impaired mitochondrial function in Leydig cells, causing oxidative stress. The oxidative stress initiated the apoptotic process through the BAX/BCL2 pathway, further inhibiting testosterone levels and steroid synthesis.

## 5. Conclusions

**Figure 6:**
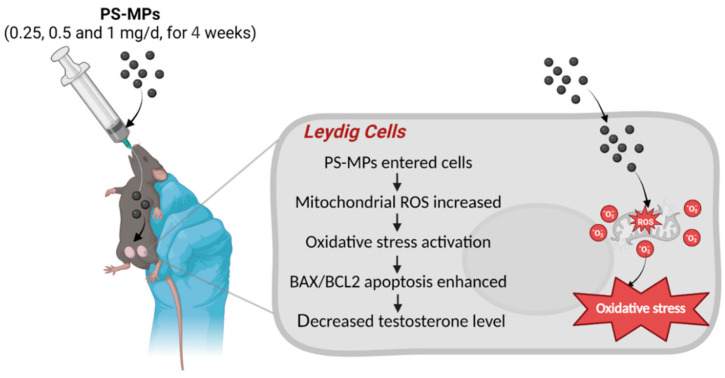
Chronic exposure to polystyrene microplastics induces decreased testosterone levels in mice via activated mitochondrial oxidative stress and BAX/BCL2 apoptosis. PS-MPs refers to polystyrene microplastics; ROS to reactive oxygen species; BAX to BCL2-associated X protein; and BCL2 to BCL2 apoptosis regulator.

In summary, through in vivo and in vitro studies, we confirmed the toxicity of chronic exposure to MPs on testosterone levels in male mice. We proposed that this damage is related to mitochondrial oxidative stress and BAX/BCL2-mediated apoptosis in Leydig cells (Figure 6). Our results provide a basis for future studies on the effects of MPs on male reproductive health and offer new insights into the health risks posed by MPs.

## Data Availability

The datasets generated during and/or analyzed during the current study are available from the corresponding author on reasonable request.

## Supplementary Materials

The following supporting information can be downloaded at https://www.mdpi.com/article/10.3390/toxics12080561/s1: Figure S1: Characteristics and images of PS-MPs; Figure S2: Effects of PS-MPs on cell viability in testicular Leydig cells; Table S1: Oligonucleotide primers for RT-qPCR.
