# Supplementary material for: Chronic Polystyrene Microplastic Exposure Reduces Testosterone Levels in Mice through Mitochondrial Oxidative Stress and BAX/BCL2-Mediated Apoptosis
Source: Toxics. 2024 Aug 1;12(8):561. doi: 10.3390/toxics12080561 (PMC11359750; doi:10.3390/toxics12080561)
Supplement: Supplementary file 1 [file toxics-12-00561-s001.zip › toxics-3104490-supplementary.pdf]

S1. Characteristics and images of PS-MPs

The morphology of PS-MPs used in this study was determined with a SEM (Fig. S1A). The PS-MPs exhibited a spherical shape with uniform dimensions. According to the Raman spectrum (Fig. S1B), the monomer of MPs was polystyrene. The PS-MPs in the testes were observed with TEM, and the red arrows indicated PS-MPs was ingested and degraded in Leydig cells (Fig. S1C).

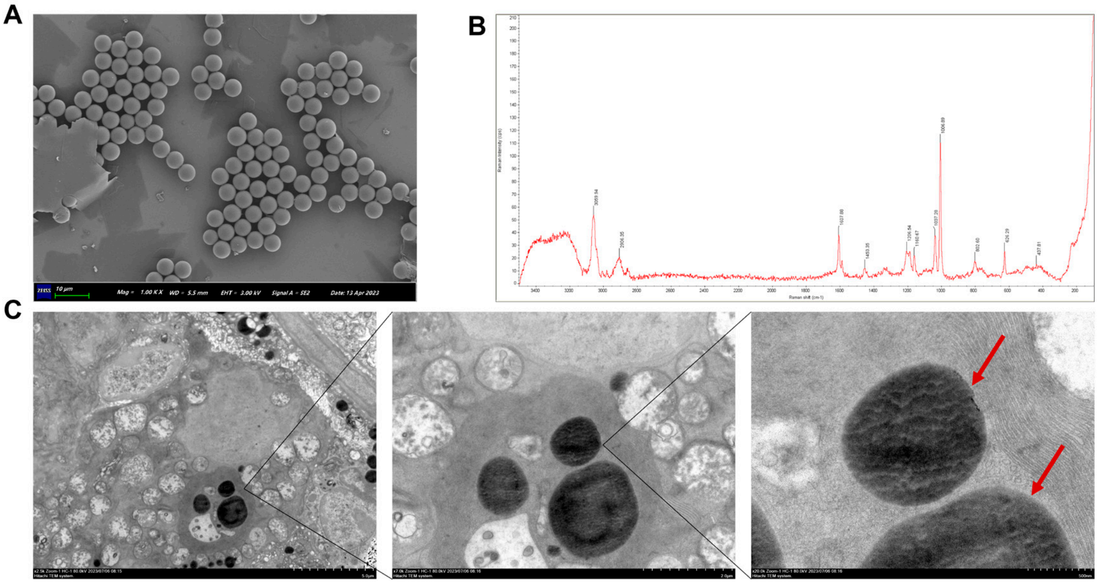

Figure S1. Characteristics and images of PS-MPs. (A) Morphological characterizations of PS-MPs by SEM; (B) Raman spectrum characterizations of PS-MPs; (C) The distribution of PS-MPs in testis by TEM. PS-MPs, polystyrene microplastics; SEM, scanning electron microscope; TEM, transmission electron microscope.

S2. Effects of PS-MPs on cell viability in testicular Leydig cells

Firstly, cell viability was detected by MTS assay, and the results showed that none of the treated groups had significant differences except on 72h PS-MPs treatment (Fig. S2A-C)

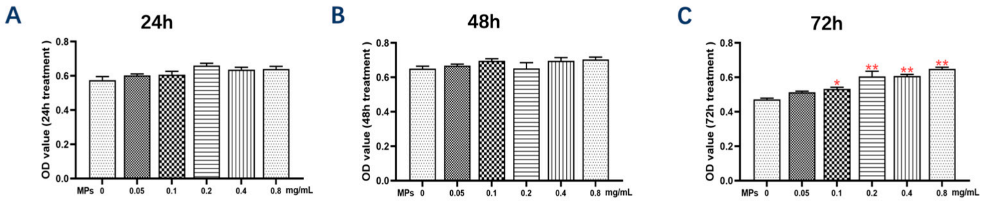

Figure S2. Effects of PS-MPs on cell viability in testicular Leydig cells. (A-C) Cell viability detection by MTS assay (n=6). Means ± S.E.M., $^{*}P<0.05$, $^{**}P<0.01$ *vs.* control. PS-MPs, polystyrene microplastics.

Table S1. Oligonucleotide primers for RT-qPCR.

Species	Genes	Forward primer	Reverse primer	Annealing (°C)
Mouse	GAPDH	TCTCCTGCGACTTCAACA	TGTAGCCGTATTCATTGTCA	60(30 s)
	StAR	AACTGGAAGCAACACTCTAT	GGCACCATCTTACTTAGCA	60(30 s)
	P450scc	TTCATCAATGCTGTCTACCA	AGTCTCGCTTCTGCCTTA	60(30 s)
	HSD3b1	TGCCAGCCTTCATCTTCT	CTGCCAGCACTGCCTTCT	60(30 s)
	CYP17a1	TTGGAAATGATAAAGGAAC	TTGGCTTGTATCAGAATGT	60(30 s)
	BCL2	TGGAGAGCGTCAACAGGGAG	GCCAGGAGAAATCAAACAGA	60(30 s)
	BAX	CCTTTTTGCTACAGGGTTTC	TTGCTGTCCAGTTCATCTCC	60(30 s)

RT-qPCR, real-time quantitative polymerase-chain-reaction; GAPDH, glyceraldehyde 3-phosphate dehydrogenase; StAR, steroidogenic acute regulatory protein; P450scc, cytochrome P450 cholesterol side chain cleavage; HSD3b1, hydroxysteroid 3-beta dehydrogenase 1; CYP17a1, cytochrome P450 family 17 subfamily A member 1; BCL2, BCL2 apoptosis regulator; BAX, BCL2 associated X, apoptosis regulator.
